# Comparative Evaluation of Spectroscopic Sensor Modalities (LIBS, MIRS, and VNIR–SWIR Hyperspectral Imaging) for the Quantification of Calcium Carbonate

**DOI:** 10.3390/s26092609

**Published:** 2026-04-23

**Authors:** Assaad Kanaan, Josette El Haddad, Paul Bouchard, Christian Padioleau, Francis Vanier, Aïssa Harhira, François Vidal

**Affiliations:** 1 Centre Énergie Matériaux Télécommunications, Institut National de la Recherche Scientifique, 1650 Lionel-Boulet Blvd, Varennes, QC J3X 1P7, Canada; 2 National Research Council of Canada, 75 de Mortagne Blvd, Boucherville, QC J4B 6Y4, Canada; josette.elhaddad@cnrc-nrc.gc.ca (J.E.H.); paul.bouchard@cnrc-nrc.gc.ca (P.B.); christian.padioleau@cnrc-nrc.gc.ca (C.P.); francis.vanier@cnrc-nrc.gc.ca (F.V.); aissa.harhira@cnrc-nrc.gc.ca (A.H.)

**Keywords:** carbon detection, CaCO_3_-CaO mixtures, sensing performance, laser induced breakdown spectroscopy, mid infrared spectroscopy, hyperspectral imaging, short-wave infrared, visible/near-infrared, multivariate analysis, partial least squares regression

## Abstract

**Highlights:**

**What are the main findings?**

- A comparative evaluation of calcium carbonate quantification, based on a number of optical spectroscopic sensor modalities (LIBS, MIRS, HSI-SWIR, HSI-VNIR) and supported by chemometrics methods to ensure robust and reproducible results, is performed.
- MIRS and HSI-SWIR deliver acceptable quantification performance. However, LIBS achieves the highest prediction accuracy compared to other techniques.

**What are the implications of the main findings?**

- This technical comparative study will assist the carbon mineralization industry in selecting the most suitable optical spectroscopic tools for real-time carbonate monitoring and product control.
- The fast and accurate quantification of carbonate produced by carbon capture enables reliable estimation of carbon credits in CO_2_-to-carbonate capture processes.

**Abstract:**

This study presents a comparative evaluation of multiple-approach optical spectroscopic sensor—Laser-Induced Breakdown Spectroscopy (LIBS), Mid-Infrared Spectroscopic sensing (MIRS), and Hyperspectral Imaging (HSI)-based sensors operating in the Visible–Near-Infrared (VNIR) and Short-Wave Infrared (SWIR) ranges—for the quantitative detection of calcium carbonate (CaCO_3_) in pelletized CaCO_3_-CaO mixtures. The objective was to assess and compare the sensing performance of these optical sensor platforms for carbonate quantification. Each spectroscopic sensor dataset was processed using chemometric calibration methods, including Partial Least Squares Regression (PLSR), to ensure robust and reproducible quantitative predictions. Although the samples consisted of binary CaCO_3_-CaO mixtures, the sensing task focused exclusively on CaCO_3_ content. Results indicate that LIBS, MIRS, and HSI-SWIR-based sensing approaches achieved comparable quantitative performance, with LIBS providing the highest prediction accuracy. In contrast, the HSI-VNIR sensor configuration demonstrated lower predictive capability relative to the other optical sensing modalities. These findings highlight the potential and limitations of different optical sensor technologies for carbonate detection in heterogeneous mineral systems.

## 1. Introduction

The rapid increase in global CO_2_ emissions—driven mainly by energy production, transportation and by energy-intensive industrial sectors such as cement, steel, and lime production—continues to accelerate global warming and contribute to the rise in global mean surface temperature [1,2,3]. These trends have intensified the need for scalable and economically viable mitigation strategies, particularly as industries face growing financial pressures from carbon pricing mechanisms and decarbonization policies [4,5]. Among the various approaches under development, mineralization-based CO_2_ capture and sequestration has emerged as a promising pathway due to its reliance on abundant natural materials and the formation of thermodynamically stable carbonates [6,7]. In parallel, the mineralization of mine wastes has gained significant attention as a low-cost and large-scale route for permanent CO_2_ sequestration, leveraging the natural reactivity of ultramafic and industrial alkaline residues [8]. In the rapidly developing field of carbon capture technologies, the reversible conversion between CaCO_3_ and CaO (CaCO_3_ ↔ CaO + CO_2_) is a key mechanism for CO_2_ absorption [9]. Moreover, accurate quantification of calcium carbonate from calcium oxide is increasingly recognized as a key requirement for Monitoring, Reporting, and Verification (MRV) frameworks, enabling the generation of certified carbon credits in carbonate processes of capturing CO_2_ into a stable form [10]. On the other hand, other industries, such as the cement and lime industries, require analysis of CaCO_3_ and CO contents during the calcination reaction, which releases CO_2_ [11]. The conversion of CaCO_3_ to CaO directly impacts energy efficiency, product quality, and costs [12]. Beyond industrial applications, understanding the proportions and transformations of these compounds is crucial in geology and archaeology for characterizing mineral formations [13], assessing the degradation of historic structures [14], and understanding ancient building materials [15]. In essence, the ability to rapidly and accurately quantify CaCO_3_ in mixtures is essential for process optimization, quality control, environmental sustainability, and basic research.

Traditionally, carbonate analysis relies on wet chemistry and combustion-based techniques performed by accredited laboratories, which, although effective, are often time-consuming, labor-intensive, and destructive [16,17]. Spectroscopic methods offer substantial advantages for material characterization on-site and real-time in general and are particularly well-suited for analyzing CaCO_3_-CaO mixtures due to their distinctive optical and chemical signatures. These techniques are non-destructive or nearly non-destructive in the case of Laser-Induced Breakdown Spectroscopy [18,19], preserving the integrity of valuable or limited samples—an essential requirement for archaeological artifacts, rare geological specimens, and continuous industrial monitoring. They also provide rapid results, often in real time or within minutes, enabling fast decision-making for quality control, process optimization (e.g., monitoring calcination in cement kilns), and high-throughput screening of materials. Furthermore, spectroscopic approaches typically offer high sensitivity and selectivity, allowing the detection and quantification of small amounts of individual components within complex mixtures and, crucially, enabling clear discrimination between chemically distinct compounds such as CaCO_3_ and CaO, even though they share the same base element (calcium).

This study aims to provide a comparative evaluation of three optical spectroscopic sensor modalities—Laser-Induced Breakdown Spectroscopy (LIBS), Mid-Infrared Spectroscopic sensing (MIRS), and Hyperspectral Imaging (HSI)—with a focus on their sensing capability for the analysis of CaCO_3_-CaO mixtures, while quantitative measurements are performed specifically on CaCO_3_ content. Each optical sensor platform relies on a distinct detection principle and signal generation mechanism, making it essential to assess their sensing performance in calcium-based materials to determine the most suitable sensor configuration for targeted applications.

LIBS is a powerful atomic emission spectroscopy technique [20,21,22] that has gained widespread popularity due to its ability to provide real-time, in situ and nearly non-destructive analysis of a wide variety of materials. LIBS works by focusing a laser pulse onto the surface of a sample, causing it to ablate and form a localized plasma [23,24]. This plasma then emits light that is characteristic of the elements present in the sample [25]. By analyzing the emitted light, LIBS can determine the elemental composition of the material. One of the key advantages of LIBS is its ability to perform rapid and direct measurements without requiring prolonged sample preparation [26,27], making it particularly suited for on-site [28] or remote analysis [29]. In CaCO_3_-CaO mixture analysis, LIBS can effectively detect the elemental components of calcium carbonate, such as calcium (Ca) and carbon (C). Its real-time analysis capability is invaluable in applications requiring quick decision-making, such as environmental monitoring or industrial quality control [30,31].

MIRS operated either in the transmission or the reflection modes [32,33,34], is another widely used spectroscopic technique, valued for its ability to identify and characterize the molecular structure of a variety of compounds. MIRS operates by measuring the absorption of mid-infrared radiation (typically 2.5–25 µm) by a sample, which excites fundamental molecular vibrational modes associated with specific chemical bonds [35,36]. These transitions are unique to the chemical bonds within the molecules, allowing for identification of the sample’s composition. In the case of carbonates, MIRS can detect the characteristic molecular vibrational modes [37,38], making it an ideal technique for analyzing CaCO_3_-CaO mixtures. Recently, reflection-mode MIR spectroscopy using tunable quantum cascade lasers (QCLs) has emerged as a powerful alternative, offering high spectral brightness, narrow linewidths, and enhanced surface sensitivity, which make it particularly suitable for in situ carbonate characterization [39,40].

Finally, HSI is a powerful remote sensing technique that enables the capture of images containing detailed spectral information distributed spatially across a wide range of wavelengths, typically from the visible spectrum into the near-infrared (VNIR, 0.4–1 µm) and short-wave infrared (SWIR, 1–2.5 µm) regions [41,42,43,44]. HSI records the intensity of light across hundreds of distinct wavelength bands, creating a spectral signature for each pixel in the image [45]. This spectral data can then be used to identify and map materials based on their chemical composition. The primary advantage of HSI in the analysis of CaCO_3_-CaO mixtures lies in its ability to provide spatially resolved spectral data, allowing for the visualization of carbonate mineral distributions within a sample or across a broader landscape [46]. This capability makes HSI particularly valuable in applications such as geological mapping, precision agriculture, and environmental monitoring [47]. The ability to detect subtle variations in mineral content is especially important when traditional methods struggle to differentiate between closely related carbonate compounds [48].

In this study, PLSR was employed to extract quantitative information from the acquired spectra, with calibration models developed to predict the CaCO_3_ content in CaCO_3_-CaO mixtures. The samples were prepared using controlled proportions of both components, providing a well-defined compositional framework for a rigorous comparison of the different spectroscopic sensing modalities. Model performance was evaluated using standard statistical metrics, including the coefficient of determination (R^2^), the root mean square error (RMSE), and the ratio of performance to deviation (RPD).

## 2. Materials and Methods

### 2.1. Sample Preparation

**Figure 1:**
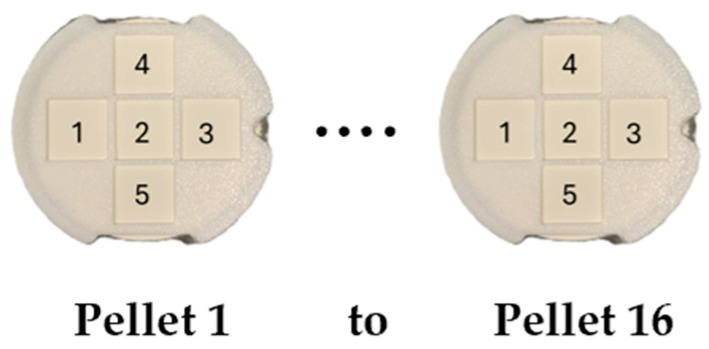
Pellets 1 and 16, containing pure CaCO_3_ and pure CaO, respectively, obtained by compression of their powder. The numbers 1 to 5 indicate the areas (S1 to S5) where the spectra were taken. All the pellets were covered with 3D-printed layers, pierced with square holes forming similar areas 1 to 5.

Sixteen circular pellets 4 cm in diameter were prepared by mixing thoroughly and then compressing 8 g of powders with different weight ratios of CaCO_3_ and CaO under a force of 15 tons for four minutes. The percent by weight (% *w*/*w*) of CaCO_3_ and CaO in each pellet is shown in Table 1, together with the role of the pellet (calibration or test) in the quantitative analysis. Five areas were identified on each pellet, hereafter referred to as S1 to S5, where average spectra were collected. Thus, for each sample, five average spectra were obtained. In total, the dataset of 16 samples contains 16 × 5 = 80 average spectra. Figure 1 illustrates Pellets 1 and 16 (pure CaCO_3_ and pure CaO, respectively), covered by 3D-printed layers, enabling accurate positioning and consistent selection of the five predefined measurement areas on each sample, and ensuring reproducibility across the four optical diagnostics. Although the samples are visually indistinguishable, the optical techniques used in this study reveal significant differences in composition.

The pellets used in this study represent idealized samples, but such grinding, homogenization, and pelletization steps are routinely applied in industrial quality-control workflows to minimize variability before spectroscopic analysis. Using standardized pellets allowed us to compare the intrinsic performance of LIBS, MIRS, and HSI-SWIR under controlled and reproducible conditions, without confounding effects from particle size, moisture, or surface roughness.

### 2.2. Experimental Setups

MIRS was performed first to probe the molecular vibrational characteristics of the samples, followed by hyperspectral imaging in the SWIR range and subsequently in the VNIR range. LIBS measurements were conducted last to provide elemental information and to ensure that each technique was performed on a flat sample surface. Indeed, the LIBS process involves ablation, which locally removes material from the pellet surface. For this reason, LIBS was intentionally performed at the end of the measurement sequence to avoid altering the sample prior to MIRS and HSI acquisition.

#### 2.2.1. LIBS Setup

**Figure 2:**
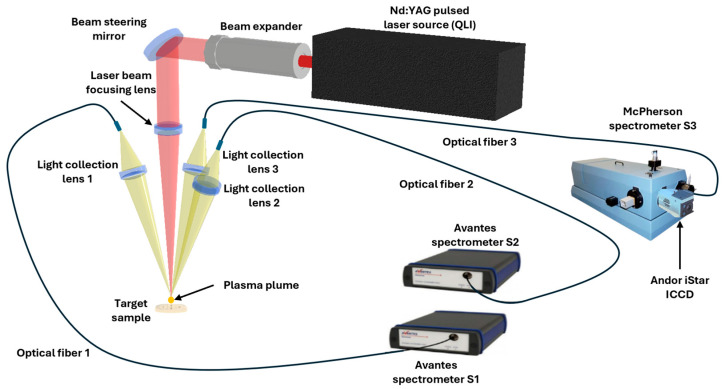
Schematic of the setup used for LIBS measurements.

The setup schematized in Figure 2 was employed for the LIBS analysis of the samples. This system is based on a 1064 nm Nd:YAG laser (Q2HE-E50, Quantum Light Instruments, Vilnius, Lithuania) delivering pulses up to 70 mJ with a duration of 5.8 ns, focused onto the sample surface with a spot diameter of 65 µm and operating at a repetition rate up to 50 Hz. The optical configuration includes a +175 mm focal length lens for focusing and a +55 mm lens for plasma emission collection. Spectral acquisition is performed using two spectrometers (ULS2048CL-EVO, Avantes, Apeldoorn, The Netherlands): Spectrometer 1 (S1) covers the 200–447 nm range with a resolution of 270–380 pm (3-pixel width), offering high precision in the UV–visible region, while Spectrometer 2 (S2) spans from 425 nm to 920 nm with a resolution of 690–780 pm (3-pixel width), enabling comprehensive coverage of elemental emissions. Additionally, a high-resolution Czerny-Turner spectrometer (model 207, McPherson, Chelmsford, MA, USA) covers the 240.81–254.88 nm range with a spectral resolution of 37 pm at 248 nm. An iStar intensified charge-coupled device (ICCD) camera (DH720-25-03, Andor Technology, Belfast, UK) is attached to the McPherson spectrometer to enhance sensitivity for carbon detection, ensuring improved accuracy in elemental quantification and enabling precise measurement of the carbon emission line at 247.8 nm. This setup was specifically used to measure the carbon emission line at 247.8 nm. The three spectrometers recorded spectra produced by the same laser shot.

A parametric LIBS study was carried out to optimize the experimental conditions for the analysis of CaCO_3_ and CaO pellets. Five representative pellets (1, 4, 9, 14, and 16) were examined to determine the most reliable number of laser shots among 50 consecutive acquisitions taken at the same position, repeated across three positions for each sample. The average signal from the first 20 shots showed the strongest correlation with carbon content, so this number was retained for the measurements.

The protocol consisted of a 7 × 7 shot matrix for each analysis area, covering five zones per pellet, yielding a total of 4900 spectra per sample (5 × 7 × 7 × 20). Laser scans were performed with a spatial pitch of 1.3 mm between positions and a repetition rate of 1 Hz. The laser energy was 17 mJ, and a delay time of 1 µs was applied for both the McPherson and the Avantes spectrometers.

#### 2.2.2. MIRS Setup

**Figure 3:**
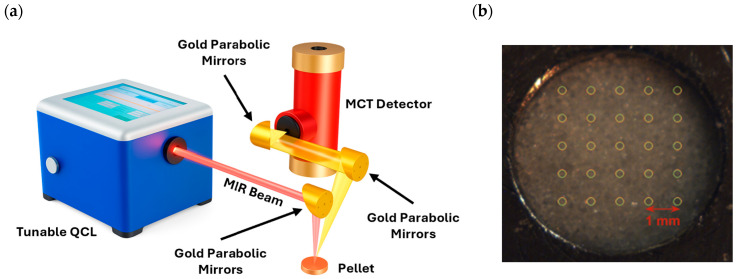
(**a**) Schematic view of the setup for the QCL-MIR reflection spectroscopy measurements. (**b**) KBr powder reference lightly pressed by hand to adjust the surface flatness, showing the position of the 25 measurements averaged for normalization. A distance of 1 mm, center to center, separates each scanned region.

Figure 3a shows the mid-infrared (MIR) reflectance spectroscopy setup based on an external-cavity quantum cascade laser (EC-QCL; LaserTune, Block Engineering, Southborough, MA, USA). The system spans a spectral range of 5.21–13.42 μm (745–1920 cm^−1^) through four integrated laser diodes with overlap regions at 970, 1344.7, and 1632.4 cm^−1^. These values correspond to diode switching points. The QCL is operated in a continuously tuning fashion, meaning that the EC-QCL grating, and thus the emission wavenumber, is continuously and linearly scanned across the spectral range. The raw MIR spectra are recorded by continuously scanning across the 745–1920 cm^−1^ spectral range at a point resolution of 0.235 cm^−1^ (5000 points over 1175 cm^−1^) while the spectral resolution of the QCL source is 2 cm^−1^. Therefore, this ensures key absorption bands are fully seen across the spectrum at a spectral resolution of 2 cm^−1^, similarly to traditional FTIR operation. More information about the MIR setup can be found in reference [40]. Since the spectral regions around the diode-overlap zones exhibit discontinuities, the following sampling points were removed from the analysis: 11 points in the 10,315.39–10,282.99 nm region, 54 points in the 7507.34–7433.89 nm region, and 55 points in the 6143.79–6093.66 nm region. The QCL delivers pulses with a duration of 100 ns at a repetition rate of 10 kHz. The laser beam is directed perpendicularly onto the sample using 1″ gold-coated off-axis parabolic mirrors with a reflected focal length of 100 mm, producing a 300 μm spot at a working distance of 100 mm. Reflected radiation is collected at 20° relative to the surface normal and focused onto a 1 × 1 mm^2^ liquid-nitrogen-cooled (77 °K) Mercury Cadmium Telluride (MCT) detector (FTIR-16, InfraRed Associates, Stuart, FL, USA) using two similar parabolic mirrors with a reflected focal length of 100 mm and 50 mm, respectively. Instrumental parameters were optimized through preliminary tests on CaCO_3_ and CaO pellets. The finalized acquisition protocol comprised a series of measurement grids applied to five distinct regions on each pellet. Each grid consisted of a 7 × 7 square matrix of measurement sites, with one laser shot per site and a spatial step size of 1 mm, totaling 245 spectra per sample. The 245 spectra were normalized with the average of 25 spectra measured on a pressed potassium bromide (KBr) pellet (Figure 3b), used as a reference due to its lack of absorption bands in the QCL spectral range.

#### 2.2.3. HSI Setup

In the present study, two hyperspectral systems were employed: a camera operating in the SWIR range (L-EOS 2.8, Photon etc., Montreal, QC, Canada) and a camera covering the VNIR range (PICA XC2, RESONON, Bozeman, MT, USA). The SWIR camera operates in the SWIR range, covering wavelengths from 0.9 to 2.8 μm (320 wavelength bands). The system is equipped with a focal plane array (FPA) of 320 × 256 pixels and a pixel size of 30 μm. Spectral data was acquired with a spectral sampling interval of 5.9 nm per pixel. Reflectance in the SWIR/V-NIR regions was determined by normalizing sample measurements to spectral data obtained from a Spectralon^®^ diffuse reflectance reference panel (Labsphere, NH, USA) from 250 to 2500 nm acquired under identical conditions. Several acquisition parameters were optimized to ensure consistent hyperspectral data across both imaging systems. For the SWIR camera, optimal performance was achieved at 50 frames per second (fps) and a stage translation speed of 50 mm/s, offering a balance between speed and spectral resolution. Similarly, the VNIR camera operates from 0.4 to 1 µm (462 wavelength bands with a spectral resolution of 1.9 nm at FWHM) at a frame rate of 30 fps, with the translation stage moving at 30 mm/s.

### 2.3. Data Treatment

To evaluate the quantitative prediction capabilities of the three spectroscopic techniques (LIBS, MIRS, and HSI), PLSR models were developed for each dataset, using a consistent modeling strategy. PLSR is a widely used linear chemometric technique that models the relationship between high-dimensional spectral data and target variables by extracting latent variables (LVs) optimally correlated with the target variables [49]. It is especially suited for datasets with collinearity. In our workflow, PLSR was first applied to the full spectral range of each technique, ensuring that all recorded wavelengths were initially considered in the calibration process.

A critical hyperparameter in PLSR is the number of LVs, which must be carefully optimized to balance model complexity and predictive performance. In this study, the number of LVs was systematically screened within a predefined range of 1 to 20, a range commonly adopted in chemometric analyses of spectroscopic datasets with similar dimensionality. From this search space, only the single optimal LV was retained for each model, based on the minimum of the RMSECV.

All PLSR calculations were performed using the PLS_Toolbox software version 9.5 (Eigenvector Research, Inc., Manson, WA, USA) within Matlab^®^ environment version R2023b (Mathworks, Natick, MA, USA).

Eleven samples were used for calibration and five for prediction, as specified in Table 1. For the quantitative analysis, the PLSR model was applied using five averaged spectra per sample (from areas S1 to S5), with 55 spectra in the calibration test and 25 spectra in the test set.

The performance of each PLSR model was evaluated using standard statistical indicators, including the root mean square errors of calibration (RMSEC), cross-validation (RMSECV), and prediction (RMSEP), the coefficients of determination (R^2^), and the ratio of performance to deviation (RPD). The RPD is defined as the ratio between the variability of the reference data and the prediction error of the model. It is generally accepted that an RPD > 2.5 corresponds to a good predictive model.

In PLSR, the root mean square error of calibration (RMSEC) was calculated from the residuals between reference and predicted values in the calibration set. Model robustness was evaluated using Venetian blinds cross-validation [50,51], in which the spectra of all samples were assigned to k folds following a cyclic modulo-k sequence (1, 2, …, k, 1, 2, …). This strategy ensures that each fold constitutes a representative subset of the overall dataset, thereby minimizing potential local bias. To prevent information leakage, replicate spectra from the same sample were distributed across different folds. At each iteration, one fold was excluded from model training and used for validation, while the model was fitted on the remaining folds. This process was repeated until every fold had served once as validation data. The resulting root mean square error of cross-validation (RMSECV) represents the aggregated prediction error across all validation folds and provides an internal estimate of model robustness. The performance of the resulting linear PLS model was validated using a set of independent test samples to avoid overfitting. The root mean square prediction error (RMSEP) was calculated from this independent test set.

These metrics offer a comprehensive evaluation of the model’s accuracy, predictive power, and generalizability. The coefficient of determination (R^2^) was calculated for calibration (R^2^ Cal), cross-validation (R^2^ CV), and prediction (R^2^ Pred) sets.

Three preprocessing methods were systematically applied to each dataset: Standardization (centering each wavelength to zero mean across the samples and scaling to unit variance to equalize feature influence), Vector normalization/L1 normalization (1-Norm, Area = 1; each spectrum was scaled such that the sum of its intensity values equals one, reducing differences in overall intensity while preserving spectral shape), and Standard Normal Variate (centering each spectrum and scaling by its standard deviation).

An 11-point second-order Savitzky–Golay filter was applied to the MIRS and HSI spectra to smooth the spectra while preserving key spectral features, reducing high-frequency noise without distorting the molecular bands of interest [52].

In addition, the Grunwald-Leitnikov Fractional Order Derivative (FOD) was applied as a preprocessing technique to enhance the spectral features and improve the performance of multivariate models [53]. Unlike conventional first- or second-order derivatives, FOD allows the adjustment of the derivative order to non-integer values, providing greater flexibility to optimize the balance between noise reduction and resolution of overlapping peaks. The choice of order from 0.5 to 5.5 with increments of 0.1 was optimized for each data set based on an evaluation of their impact on model performance. FOD is most commonly used in molecular spectroscopy. In this work, it is also applied to LIBS to complete the preprocessing comparison across different spectroscopic techniques.

It is important to note that fractional derivative orders do not correspond to specific physical transformations of the spectrum. Instead, FOD acts as a flexible mathematical operator that continuously adjusts the balance between smoothing, baseline suppression, and peak sharpening. Because these effects depend on the noise structure, baseline behavior, and spectral resolution of each modality, the optimal fractional order cannot be predicted from physical principles alone and must be determined empirically. In this study, the FOD order was therefore optimized in the same manner as other preprocessing hyperparameters (e.g., Savitzky–Golay window size), with the objective of selecting the most suitable transformation for each spectroscopic technique.

Furthermore, variable selection is applied to datasets. Variable selection is a critical step in multivariate spectroscopic modeling, as incorporating the full spectral range often introduces collinearity, amplifies noise and reduces model robustness when applied to independent samples. Two types of variable selection were applied in this study. The first variable reduction was performed using a correlation-based thresholding approach, whereby only wavelengths showing significant correlation with CaCO_3_ concentrations were retained. The applied threshold of coefficient correlation is 0.4, so the wavelength values that have a coefficient correlation between 0.4 and 1 were kept. The second variable selection was performed using the Variable Importance in Projection (VIP)-based thresholding approach [54]. The selected wavelengths of VIP values above 0.9 or 1.0 were kept. This procedure ensured that the retained variables were the most chemically informative while excluding redundant or weakly correlated features.

The VIP threshold is not a statistical significance test but a widely used chemometric criterion to retain variables contributing above the average level to the PLSR model. Because PLSR captures covariance patterns rather than isolated peak intensities, the selected wavelengths do not necessarily correspond exclusively to discrete atomic emission lines (LIBS) or to individual vibrational bands (MIRS and HSI-SWIR). Instead, VIP-based selection preserves the spectral regions, whether narrow emission features, broad molecular absorptions, or multivariate zones, that carry the strongest predictive information for the regression task. This approach ensures that the retained variables are the most informative for each modality while excluding noisy, redundant, or weakly relevant portions of the spectrum.

Although several nonlinear strategies were tested, none provided performance exceeding that of the linear PLS model. To further assess whether nonlinear regression could offer an advantage for CaCO_3_ quantification, the LIBS spectra were modeled using representative nonlinear frameworks. Despite their theoretical ability to capture complex spectral relationships, their predictive accuracy remained limited under the present data-constrained conditions. This outcome reflects a well-known characteristic of small-sample LIBS studies: when the number of calibration samples is modest relative to the spectral dimensionality, linear latent-variable methods such as PLSR, especially when combined with VIP-based wavelength selection, tend to offer superior robustness, stronger regularization, and more stable generalization. In this work, the optimized PLSR-VIP pipeline provided the most reliable CaCO_3_ quantification, confirming that linear chemometric strategies remain highly competitive, and often preferable, when dataset size does not allow nonlinear models to fully exploit their representational capacity.

## 3. Results

### 3.1. Spectra Features

#### 3.1.1. LIBS Spectra

**Figure 4:**
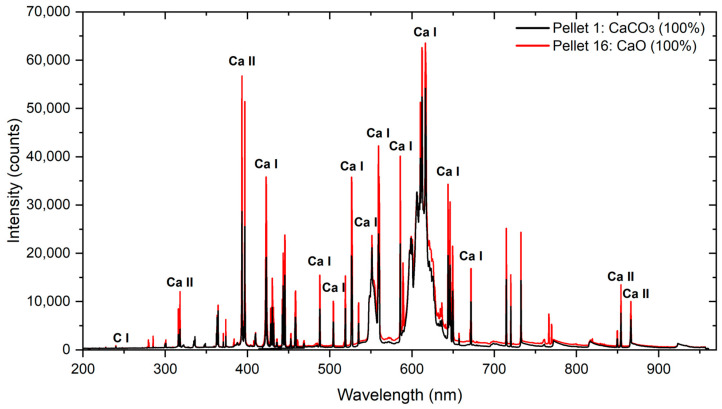
Mean spectra obtained by LIBS with low-resolution spectrometers (Avantes) for CaCO_3_ and pure CaO pellets (Pellet 1 and Pellet 16, respectively).

**Figure 5:**
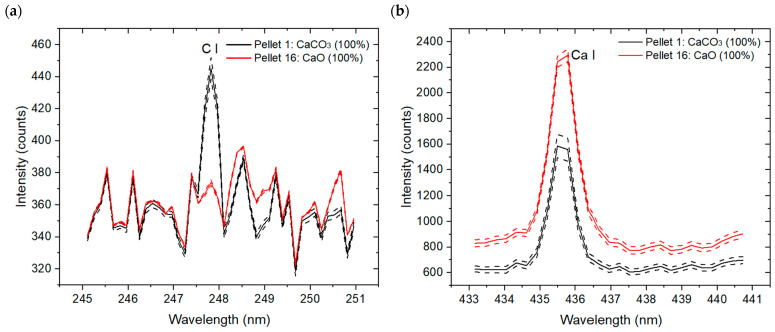
LIBS mean spectra from Avantes spectrometers for Pellet 1 (pure CaCO_3_) and Pellet 16 (pure CaO). Two lines are identified: (**a**) a carbon line at 247.85 nm, and (**b**) a calcium line at 435.5 nm. The dashed spectra represent the ± standard deviation (SD) from the five areas shown in Figure 1.

The LIBS mean spectra of pure CaCO_3_ (Pellet 1) and pure CaO (Pellet 16) from the two Avantes spectrometers shown in Figure 4, exhibit similar spectral structures over the 190–960 nm range, particularly in Ca emission lines throughout the entire spectrum and in the CaO molecular band emission observed between 590 nm and 630 nm. Despite this overall similarity, differences in emission features allow reliable discrimination between the two compounds. The CaO pellet shows systematically stronger Ca I and Ca II emission lines, reflecting the more direct release of calcium atoms from the oxide matrix during plasma formation. A zoomed-in view of the Ca line is shown in Figure 5. Although oxygen is present in CaO and CaCO_3_, the most intense O I atomic emission lines, near 777 nm, are not clearly resolved in either case, as they are partially masked by the intense CN molecular bands formed during plasma–air interactions and carbonate decomposition. This is a consequence of the use of a reduced laser fluence to avoid the saturation of the intensities of the Ca lines. In contrast, the CaCO_3_ pellet exhibits a weak but clearly detectable C I emission line, originating from the carbon contained in the carbonate structure. These spectral characteristics highlight the influence of matrix-dependent plasma chemistry and demonstrate the capability of LIBS to effectively discriminate between calcium carbonate and calcium oxide.

Figure 5a,b present the C I emission at 247.85 nm and the Ca I line at 435.5 nm, respectively, of Pellets 1 and 16. Dashed curves represent the standard deviation of the five surface measurement zones (S1–S5). These envelopes indicate the variability around the mean signal and the repeatability of LIBS measurements. The narrow spacing between the mean spectra and the envelopes confirms the pellets’ spatial homogeneity, adequate mixing procedure, and the robustness of the acquisition protocol. The C I emission line is observed only in CaCO_3_, while the Ca I line intensity is higher in CaO than in CaCO_3_.

**Figure 6:**
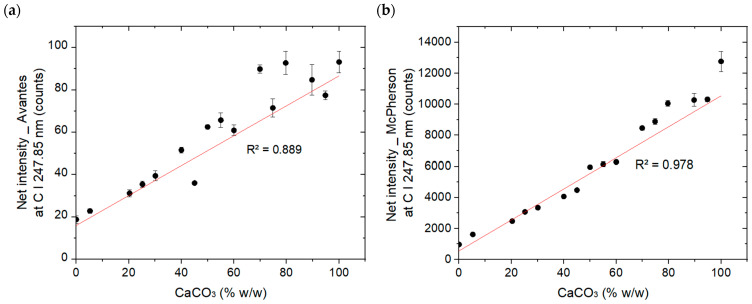
LIBS mean net intensity at carbon line C I (247.85 nm) obtained (**a**) with the Avantes spectrometer and (**b**) with the McPherson spectrometer vs. the CaCO_3_ (% *w*/*w*) content. Error bars represent standard deviations of the five surface measurement zones (S1–S5).

Figure 6 shows the net average intensity (peak minus nearby background) of the 247.85 nm carbon emission line with (a) Avantes spectrometer 1 and (b) higher resolution McPherson spectrometer for 16 pellets versus the CaCO_3_ content (% *w*/*w*). The net intensity was determined after performing adjacent background subtraction. Specifically, the background was estimated by averaging the signal intensity within the spectral range of 247.01–247.50 nm, and this value was subtracted from the measured peak intensity at 247.8 nm. The figure highlights the quantitative relationship between the spectral response and the carbonate content.

As shown in Figure 6b, the net carbon peak intensity increased progressively with increasing the CaCO_3_ content, reaching a maximum of 12,763.12 counts at 100% CaCO_3_ content. The minimum net intensity of 972.70 counts was observed in the 0% CaCO_3_ content (100% CaO content). The presence of the carbon line, in the absence of CaCO_3_, is attributed to carbon originating from CO_2_ in the ambient atmosphere. Since the Avantes spectrometer produced a low signal intensity even at 100% CaCO_3_ content, the coefficient of determination is lower (Figure 6a, R^2^ = 0.889) than that of the McPherson spectrometer, which provides a high intensity signal (Figure 6b, R^2^ = 0.978). Consistently, the reproducibility of the measurements between the five areas S1 to S5 is higher for the high-resolution McPherson spectrometer (RSD = 2.95%) compared to the lower resolution Avantes spectrometer (RSD = 4.69%). Based on these results, we can conclude that matrix effects between CaO and CaCO_3_, plasma temperature fluctuations, and self-absorption of the C I 247.8 nm line were minor under our experimental conditions. The homogeneity of the samples and the similar behavior of the two matrices ensured stable and reproducible plasma conditions. Therefore, these effects should not affect the performance or applicability of the linear PLS regression method in future carbonate quantification studies.

#### 3.1.2. MIR Spectra (746.6–1918 cm^−1^)

Prior to analysis, all MIR spectral data underwent a preprocessing stage to improve signal quality and comparability. The detector baseline was first subtracted from the raw signals. This was accomplished by blocking the QCL laser beam and recording the detector output under identical acquisition settings. Spectra intensity was then normalized relative to the KBr spectrum, which serves as a non-absorbing reference matrix in the mid-infrared region to reveal the reflectance spectrum. This normalization corrects for the experimental setup influences on the spectra, such as the spectral intensity of the QCLs, the absorption of air and the spectral response of the detector. The processed spectra then represent specifically the intrinsic reflectance features of the samples, thereby improving comparability and spectral quality. Finally, a Savitzky–Golay filter was applied to smooth the spectra [52]. Considering that the raw MIR spectra are at a point resolution of 0.235 cm^−1,^ with a QCL source spectral emission line resolution of 2 cm^−1^.

**Figure 7:**
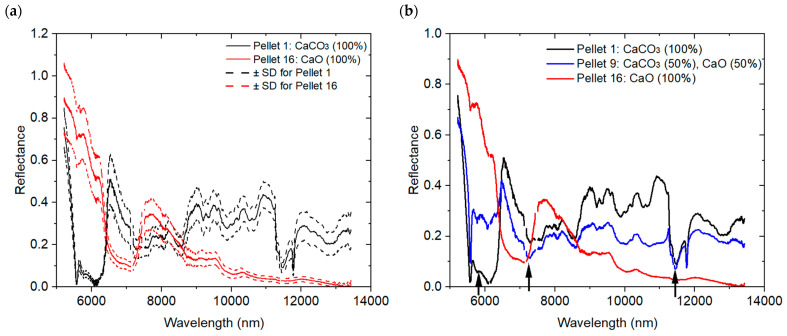
Processed MIR mean reflectance spectra. (**a**) Intra-sample variability illustrated by a dashed envelope derived from measurements acquired over five areas (S1–S5) of Pellets 1 and 16; (**b**) Inter-sample variability among Pellets 1, 9, and 16. Arrows in (**b**) indicate absorption bands of CaCO_3_ near 5900 nm, 7100 nm and 11,500 nm (1694.92, 1408.45, and 869.57 cm^−1^).

Figure 7 presents processed MIR spectra illustrating both intra- and inter-sample variability. In Figure 7a, two mean spectra are shown, corresponding to Pellets 1 and 16. Each mean spectrum was obtained by averaging measurements acquired over the five analyzed areas (S1 to S5) of the corresponding pellet. The dashed curves forming an envelope around each mean spectrum represent the dispersion of the individual spectra measured at these five areas, highlighting intra-sample variability. The relative standard deviation of Pellet 16 is 12.6% and 2.07% at the arbitrarily chosen wavelengths 5777 nm and10,000 nm, respectively.

Figure 7b shows the average MIR reflectance spectra for Pellets 1, 16, and 9, corresponding respectively to pure CaCO_3_, pure CaO, and a mixture with similar proportions. Significant spectral differences are observed between the three pellets, reflecting inter-sample variability. Carbonate vibrational bands are clearly observed in the CaCO_3_ reflection spectra around 11,500 nm (~870 cm^−1^, ν_2_ out-of-plane bending mode), 7000 nm (1400–1500 cm^−1^, ν_3_ asymmetric C–O stretching mode) and near 5900 nm, corresponding to combination or overtone bands.

Key characteristics and fundamental vibrational bands of solid carbonates are found in the 1000–1100 cm^−1^ and 1400–1600 cm^−1^ regions. Additional fundamental features located between 700 and 760 cm^−1^ can provide further insight into carbonate species, if needed, as reported in references [55,56]. Features at higher energies covered by traditional FTIR (2000 cm^−1^ to 4000 cm^−1^) are overtones and usually provide a weaker reflectance response than the fundamental bands covered by the QCL spectral range.

#### 3.1.3. HSI Spectra in SWIR (900–2800 nm) and VNIR (400–1000 nm) Ranges

**Figure 8:**
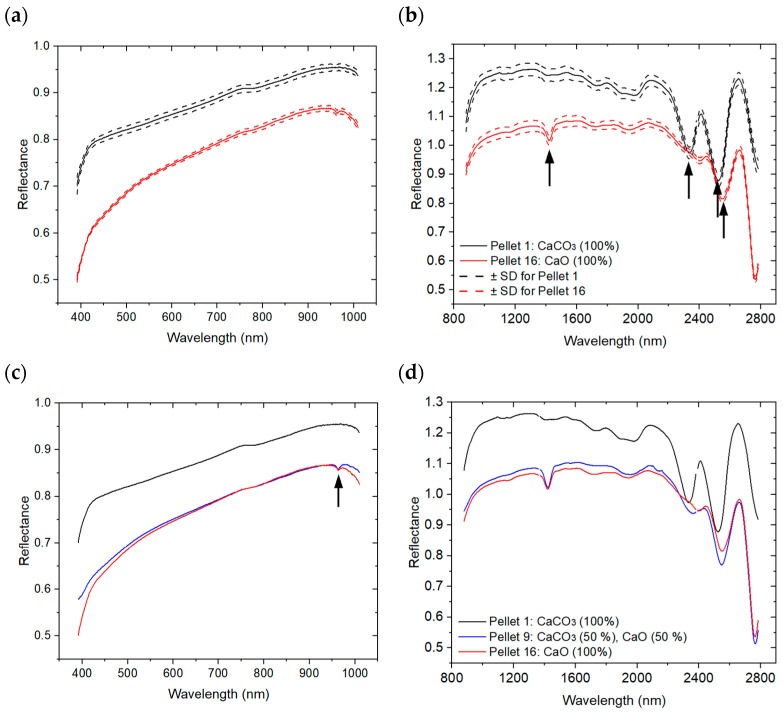
Mean HSI reflectance spectra. (**a**,**b**) Intra-sample variability of reflectance spectra illustrated by a dashed envelope derived from measurements acquired over five areas (S1–S5) of Pellet 1 and Pellet 16 in the VNIR and SWIR ranges, respectively; (**c**,**d**) Inter-sample variability among Pellets 1, 9, and 16 in the VNIR and SWIR ranges, respectively. Arrows in (**b**) indicate absorption bands attributed to Ca(OH)_2_ near 1452 nm and 2550 nm, and to CaCO_3_ near 2333 nm and 2520 nm. The arrow in (**c**) indicates an absorption band attributed to an O-H vibration near 960 nm.

Figure 8 presents the HSI measurements acquired for Pellets 1 and 16, with graph (a) corresponding to the VNIR range and graph (b) to the SWIR range. For each pellet, the mean spectrum was obtained by averaging the measurements collected over the five analyzed areas (S1–S5), while the dashed curves define an envelope around each mean spectrum and illustrate the intra-sample spectral variability. As with MIR spectra, Savitzky–Golay filtering was used to smooth HSI spectra.

Reflectance spectra of carbonate minerals have been extensively reported in the VNIR and SWIR regions [57,58], with diagnostic absorption features linked to the vibrational modes of the carbonate ion (${CO}_{3}^{2-}$). In the SWIR domain, particularly within the 2.10–2.60 μm wavelength range, carbonate minerals typically exhibit two prominent reflectance bands centered near 2300–2350 nm and 2500–2550 nm [59,60,61], whose exact positions depend on the strength of the cations’ bond to the ${CO}_{3}^{2-}$ anion [55].

Consistent with previous studies, the SWIR spectra of Pellet 1 exhibit well-defined reflectance features at 2333 nm (4285 cm^−1^), attributed to the ν_3_ + ν_4_ combination band, and near 2520 nm (3968 cm^−1^), corresponding to higher-order combination bands involving the asymmetric stretching mode ν_3_ [62]. The coefficient of variation (CV) at 2333 nm was 0.032%, indicating excellent spectral reproducibility. In contrast, Pellet 16 displays a weak absorption feature at 1452 nm (6887 cm^−1^), consistent with the first overtone (2ν) of O–H stretching vibrations, suggesting the presence of calcium hydroxide Ca(OH)_2_ [63]. This phase likely formed via the hydration of CaO upon exposure to atmospheric moisture (CaO + H_2_O → Ca(OH)_2_). The feature observed near 2520 nm in pellet 16 may partly arise from Ca(OH)_2_, as combined O–H stretching and bending vibration bands are known to occur within the 2400–2550 nm range [62].

For the VNIR range, the small reflectance feature observed near 960 nm for Pellet 9, as shown in Figure 8c, is primarily associated with water absorption (second overtone of the O-H vibration) or the presence of Ca(OH)_2_ rather than carbonate-related vibrational modes. There is no spectral feature detection related to carbonate minerals. Therefore, the VNIR range was not considered for quantitative analysis of carbonate in the following quantitative study. LIBS, MIRS and HSI-SWIR show a feature detection of carbonate directly (molecular bands) or indirectly (element emission), compared to no feature detected by VNIR.

### 3.2. Predicting CaCO_3_ Concentrations from Spectra

The spectral datasets acquired using the three sensing modalities were correlated with the carbonate content of the pellets. Table A1 in Appendix A presents the results of various combinations of preprocessing methods for replicate-level predictions. Table 2 summarizes the best-performing quantitative model for each dataset, including the optimal preprocessing strategy, allowing direct comparison of predictive accuracy and robustness. Performance metrics (RMSEP, R^2^(Pred), and RPD) were computed from the replicate-level prediction (second-to-last row) and the sample-level prediction (last row), obtained by averaging predictions of five replicates per test sample. The model was developed using replicate data, and the optimization of parameters was based on performance at the replicate level. However, for sample prediction, the average of the five replicates was used. Therefore, the following comparison between the techniques is presented at the sample level on the test set, focusing on predictive accuracy for the sample rather than replicate variability. This approach provides a more reliable and statistically meaningful assessment of the model’s overall performance.

For LIBS, univariate analysis based on the carbon emission line at 247.8 nm acquired with the high-resolution McPherson spectrometer yielded strong predictive performance (R^2^(Pred) = 0.987; RMSEP ≈ 5%). When applied to lower-resolution Avantes spectra, univariate modeling showed reduced accuracy (R^2^(Pred) = 0.872; RMSEP = 11.09%). However, multivariate PLSR applied to the Avantes LIBS spectra markedly improved performance (R^2^(Pred) = 0.990; RMSEP = 2.04%; RPD = 11.68), surpassing the univariate high-resolution approach. These results demonstrate that multivariate modeling compensates effectively for reduced spectral resolution in LIBS measurements.

**Figure 9:**
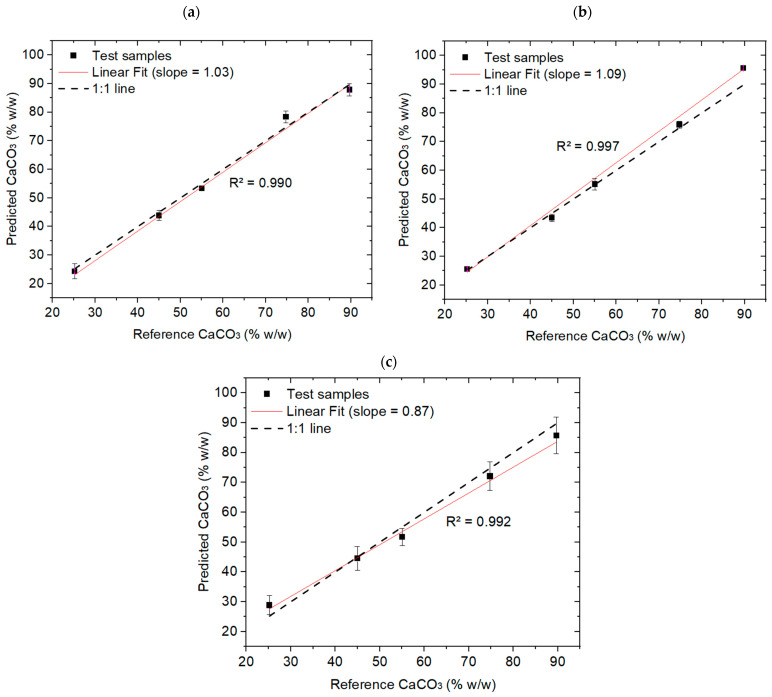
Predicted-versus-reference plots for the selected PLSR models reported in Table 2, evaluated on the independent test set: (**a**) LIBS, (**b**) MIR, and (**c**) HSI-SWIR. Sample-level predictions were obtained by averaging the predictions from five replicate spectra acquired per sample.

For MIRS and SWIR datasets, only multivariate models were considered. PLSR-MIRS achieved strong predictive capability (RMSEP = 2.78%; RPD = 8.80), slightly outperforming PLSR-SWIR (RMSEP = 3.06%; RPD = 6.65). Predicted-versus-reference plots shown in Figure 9 confirm lower replicate variability for MIRS compared to SWIR. All three sensing modalities achieved prediction errors below 5% after replicate averaging, with PLSR-LIBS providing the highest overall accuracy.

The influence of preprocessing and variable selection is displayed in Table A1. This table contains computation results for replicate-level predictions only. For LIBS, normalization significantly improved model accuracy, with SNV outperforming standardization and vector normalization. Additional FOD preprocessing provided only marginal benefits. The most substantial improvement was achieved through variable selection prior to model development. In particular, VIP-based wavelength selection (VIP > 1) applied to SNV-normalized spectra yielded the best performance (RMSECV = 2.20%, RMSEP = 2.85%) while reducing the dataset from 4096 to 308 wavelengths. These results indicate that variable selection is critical for optimizing LIBS-based quantitative models.

For MIRS, SNV normalization markedly enhanced prediction accuracy compared to standardization and vector normalization. The addition of FOD produced only minor gains. In contrast to LIBS, variable selection did not improve model performance and, in some cases, degraded it. This behavior is consistent with the broad and chemically structured vibrational features of MIR spectra, which are effectively handled by PLSR without prior wavelength reduction. The best results were obtained using a second-order Savitzky–Golay filter combined with SNV preprocessing.

For SWIR-HSI, preprocessing effects were more dataset-dependent. Standardization and vector normalization provided moderate predictive capability, whereas SNV reduced model robustness. Correlation-based variable selection was ineffective; however, VIP-based wavelength selection after normalization significantly improved model performance (RMSEP = 5.33%, R^2^(Pred) = 0.951) while reducing the number of wavelengths from 320 to 86. The optimal configuration combined second-order Savitzky–Golay filtering, vector normalization, and VIP-based variable selection.

To statistically assess whether the predictive differences between the optical sensors were meaningful, a one-way Analysis of variance (ANOVA) was performed on the individual prediction errors obtained for the 25 test spectra. The analysis revealed a significant effect of the spectroscopic technique on prediction accuracy (*p* = 0.0346 < 0.05), confirming that the observed differences are not attributable to random variation.

## 4. Discussion

Infrared spectroscopy remains a powerful tool for carbonate detection due to the presence of strong molecular absorption bands in the MIR and SWIR regions. In this study, we focused on sensor configurations compatible with industrial deployment and real-time monitoring rather than conventional laboratory FTIR systems. The results demonstrate that sensor architecture and spectral resolution play a decisive role in quantitative performance.

The VNIR hyperspectral configuration failed to detect carbonate features, confirming that this spectral range lacks the characteristic absorption bands required for CaCO_3_ quantification. In contrast, the QCL-MIR configuration provided high spectral selectivity and sensitivity due to its coherent, narrow-linewidth radiation source. Its laser-based architecture enables compact designs without interferometric components, making it attractive for industrial integration, although full spectral coverage remains costlier than conventional broadband FTIR systems.

HSI-SWIR represents a complementary approach. While its spectral resolution is lower than that of QCL-MIR, it enables rapid, large-area imaging and is therefore well suited for high-throughput process monitoring. Both QCL-MIR and HSI-SWIR detect molecular absorption features of carbonate, but QCL-MIR achieves higher quantitative accuracy, whereas HSI-SWIR provides superior spatial coverage and acquisition speed. Although the spatial resolution of HSI is higher, this study focused on the quantitative analysis of homogeneous samples, independent of point-to-point spectral variations, as the spectra were averaged prior to model calibration. Therefore, the work did not address point-to-point variability in MIR, LIBS, or HSI measurements.

LIBS offers a fundamentally different sensing principle based on elemental emission. When coupled with multivariate analysis, even a compact low-resolution configuration achieved superior quantitative accuracy. In addition to carbonate quantification, LIBS enables impurity detection and elemental screening, providing broader analytical capability. Its robustness, compactness, and tolerance to industrial environments make it particularly attractive for in-line monitoring applications.

Overall, LIBS, QCL-MIR, and HSI-SWIR exhibit complementary strengths. The optimal choice depends on industrial constraints, including required sensitivity, spatial coverage, acquisition speed, and system cost.

## 5. Conclusions

This study demonstrates that LIBS coupled with multivariate analysis (PLSR) provides the highest quantitative accuracy for CaCO_3_ determination, achieving an absolute prediction error of ~2% using a compact, low-resolution spectrometer. MIRS combined with PLSR also achieved strong performance (~2.8% error), while HSI-SWIR yielded slightly lower accuracy (~3%) but offered superior spatial imaging capability. The HSI-VNIR configuration proved unsuitable for carbonate detection due to the absence of diagnostic absorption features in this spectral range.

Preprocessing and variable selection strategies were found to be modality-dependent. Variable selection is critical for and beneficial for SWIR-HSI, whereas full-spectrum modeling is preferable for MIRS data.

Beyond analytical performance, the results highlight the industrial relevance of these sensing technologies for monitoring carbonate formation processes, including applications related to CO_2_ mineralization and carbon credit assessment. Future work should investigate the selective quantification of multiple carbonate phases in mixed systems, particularly in complex matrices derived from mine waste carbonation.

## Figures and Tables

**Table 1:** The weight concentrations of CaCO_3_ and CaO in each of the sixteen pellets. The Type indicates if the associated spectra were used in the calibration set or test set of the PLSR model.

Sample	CaO (% *w*/*w*)	CaCO_3_ (% *w*/*w*)	Type
Pellet 1	0	100	Calibration
Pellet 2	5.2	94.8	Calibration
Pellet 3	10.3	89.7	Test
Pellet 4	20.3	79.7	Calibration
Pellet 5	25.2	74.8	Test
Pellet 6	30.1	69.9	Calibration
Pellet 7	40.0	60.0	Calibration
Pellet 8	45.0	55.0	Test
Pellet 9	50.0	50.0	Calibration
Pellet 10	55.0	45.0	Test
Pellet 11	60.0	40.0	Calibration
Pellet 12	69.9	30.1	Calibration
Pellet 13	74.8	25.2	Test
Pellet 14	79.7	20.3	Calibration
Pellet 15	94.8	5.24	Calibration
Pellet 16	100	0	Calibration

**Table 2:** Summary of the best performance metrics obtained for each spectroscopic technique (LIBS, MIRS, SWIR) using their respective optimal preprocessing methods.

	LIBS (McPherson)	LIBS (Avantes)	LIBS (Avantes)	MIRS	HSI-SWIR
Pre-Processing	Net Carbon line	Net Carbon line	SNV, VIP	Savitzky–Golay (FOD, 0.8), SNV	Savitzky–Golay L1 normalization, VIP
Model	Univariate	Univariate	PLSR 7 Latent Variables	PLSR 3 Latent Variables	PLSR 8 Latent Variables
Replicate-level predictions (five replicates)	RMSEC = 6.16%	RMSEC = 10.52%	RMSEC = 1.78%	RMSEC = 1.70%	RMSEC = 5.19%
			RMSECV = 2.20%	RMSECV = 1.80%	RMSECV = 7.66%
	RMSEP = 5.34%	RMSEP = 12.09%	RMSEP = 2.85%	RMSEP = 3.05%	RMSEP = 5.33%
	R^2^ (Cal) = 0.964	R^2^ (Cal) = 0.896	R^2^ (Cal) = 0.997	R^2^ (Cal) = 0.997	R^2^ (Cal) = 0.975
			R^2^ (CV) = 0.996	R^2^ (CV) = 0.997	R^2^ (CV) = 0.945
	R^2^ (Pred) = 0.981	R^2^ (Pred) = 0.835	R^2^ (Pred) = 0.985	R^2^ (Pred) = 0.993	R^2^ (Pred) = 0.951
	RPD = 4.16	RPD = 1.93	RPD = 8.36	RPD = 8.02	RPD = 3.82
Sample-level predictions (averaging the prediction of 5 replicates)	RMSEP = 5.01%	RMSEP = 11.09%	RMSEP = 2.04%	RMSEP = 2.78%	RMSEP = 3.06%
	R^2^ (Pred) = 0.987	R^2^ (Pred) = 0.872	R^2^ (Pred) = 0.990	R^2^ (Pred) = 0.997	R^2^ (Pred) = 0.992
	RPD = 4.44	RPD = 2.11	RPD = 11.68	RPD = 8.80	RPD = 6.65

## Data Availability

The original contributions presented in this study are included in the article. Further inquiries can be directed to the corresponding authors.

## Abbreviations

The following abbreviations are used in this manuscript:

LIBS	Laser-Induced Breakdown Spectroscopy
ICCD	Intensified Charge-Coupled Device
HSI	Hyperspectral Imaging
IR	Infrared
MIRS	Mid Infrared Spectroscopy
VNIR	Visible Near-Infrared
SWIR	Short Wave Infrared
FTIR	Fourier Transform Infrared
QCL	Quantum Cascade Laser
PLSR	Partial Least Squares Regression
LV	Latent Variable
CV	Coefficient of Variation
R^2^	Coefficient of Determination
RMSEC	Root Mean Square Error of Calibration
RMSECV	Root Mean Square Error of Cross-Validation
RMSEP	Root Mean Square Error of Prediction
RPD	Ratio of Performance to Deviation
SD	Standard deviation
SNV	Standard Normal Variate
FOD	Fractional Order Derivative
VIP	Variable Importance in Projection
ANOVA	Analysis Of VAriance

## Appendix A

**Table A1:** Evaluation of the performance characteristics of the PLSR model of LIBS, MIRS and HSI-SWIR data, according to the different pre-processing methods.

		LIBS	MIRS	HSI-SWIR
	Standardization	5 Latent Variables	6 Latent Variables	7 Latent Variables
		RMSEC = 2.99%	RMSEC = 3.00%	RMSEC = 4.06%
		RMSECV = 3.76%	RMSECV = 3.61%	RMSECV = 7.79%
		RMSEP = 6.56%	RMSEP = 6.03%	RMSEP = 7.86%
		R^2^ (Cal; CV) = 0.992; 0.987	R^2^ (Cal; CV) = 0.992; 0.988	R^2^ (Cal; CV) = 0.985; 0.944
		R^2^ (Pred) = 0.952	R^2^ (Pred) = 0.979	R^2^ (Pred) = 0.923
		RPD = 3.64	RPD = 4.06	RPD = 2.59
	Vector normalization L1	4 Latent Variables	4 Latent Variables	9 Latent Variables
		RMSEC = 2.69%	RMSEC = 2.61%	RMSEC = 3.37%
		RMSECV = 2.91%	RMSECV = 3.20%	RMSECV = 6.73%
		RMSEP = 5.99%	RMSEP = 11.02%	RMSEP = 7.74%
		R^2^ (Cal; CV) = 0.993; 0.992	R^2^ (Cal; CV) = 0.994; 0.990	R^2^ (Cal; CV) = 0.989; 0.958
		R^2^ (Pred) = 0.961	R^2^ (Pred) = 0.833	R^2^ (Pred) = 0.886
		RPD = 3.98	RPD = 2.22	RPD = 2.63
Pre-Processing	Standard Normal Variate	4 Latent Variables	3 Latent Variables	6 Latent Variables
		RMSEC = 2.53%	RMSEC = 1.72%	RMSEC = 4.12%
		RMSECV = 2.74%	RMSECV = 1.81%	RMSECV = 7.53%
		RMSEP = 5.78%	RMSEP = 3.06%	RMSEP = 11.47%
		R^2^ (Cal; CV) = 0.994; 0.993	R2 (Cal; CV) = 0.997; 0.997	R^2^ (Cal; CV) = 0.984; 0.948
		R^2^ (Pred) = 0.959	R2 (Pred) = 0.993	R^2^ (Pred) = 0.78
		RPD = 4.13	RPD = 8.01	RPD = 1.77
	Fractional Order Derivative (FOD, order)	(SNV) (FOD, 2.5)	(SNV) (FOD, 0.8)	(Norm) (FOD, 1.5)
		4 Latent Variables	3 Latent Variables	4 Latent Variables
		RMSEC = 3.32%	RMSEC = 1.70%	RMSEC = 11.19%
		RMSECV = 3.63%	RMSECV = 1.80%	RMSECV = 13.68%
		RMSEP = 5.65%	RMSEP = 3.05%	RMSEP = 14.09%
		R^2^ (Cal; CV) = 0.990; 0.988	R^2^ (Cal; CV) = 0.997; 0.997	R^2^ (Cal; CV) = 0.883; 0.825
		R^2^ (Pred) = 0.981	R^2^ (Pred) = 0.993	R^2^ (Pred) = 0.785
		RPD = 4.22	RPD = 8.02	RPD = 1.44
	Variable Selection (VS) using Correlation Matrix	(SNV) (VS, Between 0.4 and 1)	(SNV) (VS, Between 0.4 and 1)	(Norm) (VS, Between 0.3 and 1)
		5 Latent Variables	4 Latent Variables	3 Latent Variables
		RMSEC = 2.15%	RMSEC = 2.34%	RMSEC = 16.18%
		RMSECV = 2.51%	RMSECV = 2.52%	RMSECV = 18.64%
		RMSEP = 4.21%	RMSEP = 3.37%	RMSEP = 21.98%
		R^2^ (Cal; CV) = 0.996; 0.994	R^2^ (Cal; CV) = 0.995; 0.994	R^2^ (Cal; CV) = 0.794; 0.718
		R^2^ (Pred) = 0.977	R^2^ (Pred) = 0.993	R^2^ (Pred) = 0.611
		RPD = 5.66	RPD = 7.26	RPD = 0.92
	Variable selection using VIP of PLSR	(SNV) 7 Latent Variables	(SNV) 6 Latent Variables	(Norm) 8 Latent Variables
		RMSEC = 1.78%	RMSEC = 2.30%	RMSEC = 5.19%
		RMSECV = 2.20%	RMSECV = 2.74%	RMSECV = 7.66%
		RMSEP = 2.85%	RMSEP = 5.26%	RMSEP = 5.33%
		R^2^ (Cal; CV) = 0.997; 0.996	R^2^ (Cal; CV) = 0.995; 0.993	R^2^ (Cal; CV) = 0.975; 0.945
		R^2^ (Pred) = 0.985	R^2^ (Pred) = 0.984	R^2^ (Pred) = 0.951
		RPD = 8.36	RPD = 4.65	RPD = 3.82
